# Magnetic Solid Phase Extraction as a Promising Technique for Fast Separation of Metallic Nanoparticles and Their Ionic Species: A Review of Recent Advances

**DOI:** 10.1155/2020/8847565

**Published:** 2020-09-11

**Authors:** Ingrid Hagarová

**Affiliations:** Comenius University in Bratislava, Faculty of Natural Sciences, Institute of Laboratory Research on Geomaterials, Mlynská Dolina, Ilkovičova 6, 842 15 Bratislava, Slovakia

## Abstract

The widespread use of silver nanoparticles (AgNPs) and gold nanoparticles (AuNPs) in a wide variety of industrial as well as medical sectors is indisputable. This leads to a new concern about their presence in various environmental compartments. Since their negative effect and potential toxicity impact have been confirmed, analytical chemists focus on the development of different procedures for their reliable detection, identification, characterization, and quantification, not only in homogenous and simple matrices but also in complex environmental matrices. However, nanoparticles and their ionic species can coexist and their toxicity may differ; therefore, novel analytical approaches are necessary to monitor not only the nanoparticles but also their ionic species. The aim of this article is to bring a review of recent works where magnetic solid-phase extraction (MSPE) procedures in connection with spectrometric methods were used for separation/preconcentration and quantification of (1) silver and gold ions in various environmental samples, (2) AgNPs and AuNPs in real water samples in the presence of various coexisting ions, and (3) both species (it means Ag ions and AgNPs; Au ions and AuNPs) in real water samples. The results presented herein show the great analytical potential of MSPE procedures in connection with spectrometric methods used in these fields and can be helpful in guiding analytical chemists who aim to work on this subject.

## 1. Introduction

Separation techniques have an irreplaceable place in current analytical chemistry. It stems from the complexity of analyzed samples, as well as (ultra)trace concentrations of many studied analytes. Among separation techniques, extractions belong to the most often used. Depending on the extraction phase being used, a sample preparation procedure can be divided into solvent-based and sorption-based extraction. Sorption-based extraction techniques include solid-phase extraction (SPE), dispersive solid-phase extraction (dSPE), solid-phase microextraction (SPME), stir bar sorptive extraction (SBSE), magnetic solid-phase extraction (MSPE), thin-film microextraction (TFME), and in-tube solid-phase microextraction (IT-SPME) [1].

Solid-phase extraction (SPE) is based on the analyte partition coefficient between the sample solution and the sorbent. In this technique, the sorbent is packed inside cartridges, syringe barrels, or microcolumns. The extraction process consists of these main steps: (1) column conditioning, (2) sample loading, (3) washing, and (4) elution. In this column arrangement, several advantages over conventional liquid-liquid extraction (LLE) can be stated, such as the achievement of higher enrichment factors (EFs), faster procedures, and less consumption of organic solvents. However, it is not free of drawbacks. The column's design, as well as the sorbent leaching, cartridge channeling, and cartridge clogging lead to the prolongation of the extraction process [2]. Therefore, in order to address these limitations, special membrane-extraction disks that possess uniformly fixed particles in an inert matrix as thin beds were developed [3]. The applied sorbents and retention mechanisms involved are similar to those of using SPE cartridges [4].

Another alternative of SPE is the so-called dispersive solid-phase extraction (dSPE), which is based on the addition of a solid sorbent to an agitated sample solution for a certain time. Afterward, the suspension is usually centrifuged, and the sedimented sorbent with analytes is separated from the solution. Finally, a suitable solvent is used to elute the analytes from the sorbent before instrumental analysis. In this arrangement, no packed devices and conditioning steps are required. In recent years, different nanoparticles and nanocomposites have been preferred for use as sorbents in dSPE procedures for the extraction of target analytes. This is due to their high surface area, high chemical activity, high adsorption capacity, fast adsorption dynamics, and good mechanical and chemical stability. The possibility for their surface functionalization is another advantage that can lead to the development of effective dSPE procedures suitable for separation/preconcentration of various analytes in a broad range of analytical applications [5]. Among nanomaterials, metallic nanoparticles, metal oxide nanoparticles, silica-based nanomaterials, and carbon-based nanomaterials have received considerable attention [6, 7]. When using nanomaterials as solid sorbents, the contact area between the target analytes and the sorbent is higher than that in traditional SPE, the equilibrium rate increases, and higher extraction yields are obtained. Some of the critical aspects of this arrangement may be centrifugation and/or filtration. As a solution to this restriction, magnetic nanomaterials (MNPs) were proposed, since they perform phase separation more conveniently by the application of an external magnetic field. In this case, the method is named a magnetic solid-phase extraction (MSPE).

## 2. Magnetic Solid Phase Extraction

A brief overview dedicated to magnetic separation was summarized by Giakiskli and Anthemidis [8]. In fact, the magnetic separation had been known for many decades. Initially, the technique was used for the removal of tramp iron and for the concentration of iron ores. Since 1849, numerous patents on magnetic separation from the United States have been issued [9]. One of the first references of using magnetic separation for biotechnological purposes was described in the work by Robinson et al. [10], published in 1973. In 1987, Wikström et al. [11] described the use of magnetically susceptible additives together with an external magnetic field to speed up the phase separation of aqueous two-phase systems. In 1996, Towler et al. [12] used manganese dioxide-coated magnetite as a sorbent for the separation of radium, lead, and polonium from seawater samples. The term of magnetic solid-phase extraction (MSPE) was first introduced by Šafaříková and Šafařík in 1999 [13]. They used a reactive copper-phthalocyanine dye, which was immobilized on fine silanized magnetite particles and magnetic charcoal for the separation and preconcentration of organic compounds with planar molecular structures (such as safranin O and crystal violet).

**Figure 1:**
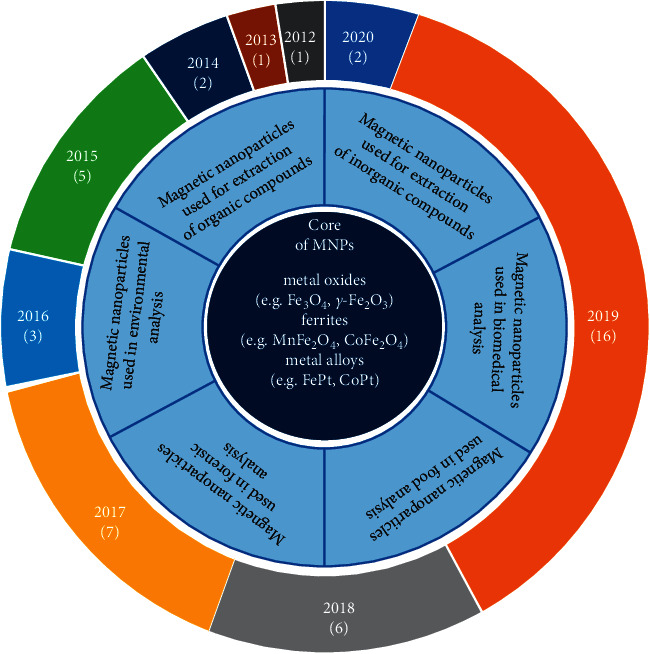
Overview of the applications of magnetic nanoparticles (MNPs) in scientific fields together with an illustration showing the number of reviews that had been annually published on this topic between January 2012 and April 2020 (according to the Web of Science database).

Since that time, diverse magnetic sorbents have been developed and used in various applications for a wide scope of sample matrices and analytes. MSPE has been extensively applied in several fields, such as biomedicine [14–18], environmental science [19–23], forensic science [24, 25], and food analysis [26–29] (Figure 1). This technique has also been successfully used for the separation/preconcentration of various types of xenobiotics (e.g., herbicides, insecticides, fungicides, aromatic and polyaromatic hydrocarbons, aromatic amines, phthalate esters, phenols and chlorophenols, aflatoxins, and heavy metal ions), as well as viruses, microbial pathogens, and protozoan parasites in environmental [30], biological, or even food samples [31].

**Figure 2:**
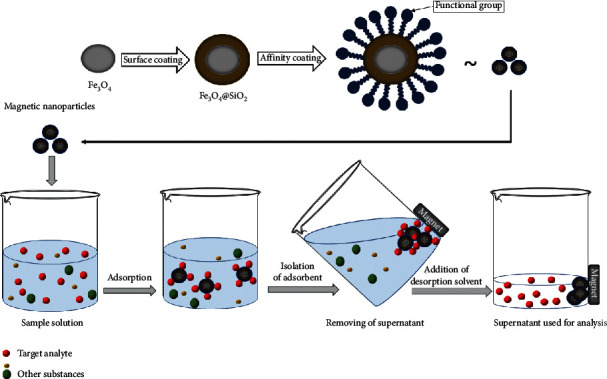
Schematic representation for the synthesis of magnetic nanoparticles (according to Liu et al. [24]) and their application in MSPE of the target analytes.

The principle of MSPE involves the addition of magnetic sorbent particles to the sample solution. The target compound is retained onto the magnetic material, and the magnetic particle (containing the analyte) is then separated from the sample solution by the application of an external magnetic field. Finally, the analyte is recovered from the adsorbent by elution with the appropriate solvent, and the eluent is then used for analysis. Magnetic adsorbents can be easily recycled and reused. A general scheme for the MSPE procedure can be found in every review published on this topic. Here, a schematic representation for the synthesis of magnetic nanoparticles (according to Liu et al. [24]) and their application in MSPE of the target analytes can be seen in Figure 2.

This approach has several advantages over traditional SPE, such as (1) faster procedures, (2) easy analyte separation with no need for centrifugation and/or filtration, (3) high selectivity of magnetic sorbents used, (4) fewer interferences since the majority of sample impurities are diamagnetic, and (5) the possibility for automation of the entire process. All these benefits lead to the development of rapid, selective, and repeatable methods for routine determinations [32]. High enrichment factors, high extraction recoveries, and acceptable precision are highlighted in all published works, thus demonstrating the great potential of MSPE procedures that can be used for (ultra)trace determinations of various analytes in a diverse variety of samples.

The aim of this paper is to bring a review of MSPE procedures used for the separation and preconcentration of silver nanoparticles (AgNPs), gold nanoparticles (AuNPs), and their ionic species. The widespread use of AgNPs and AuNPs is indisputable, resulting, therefore, in their increased concentrations in various environmental compartments. This ranks them among emerging pollutants. However, nanoparticles and their ionic species can coexist, and their toxicity may differ; novel analytical approaches are necessary to monitor not only the nanoparticles but also their ionic species. The great potential of MSPE procedures in connection with spectrometric methods (such as flame atomic absorption spectrometry (FAAS), electrothermal atomic absorption spectrometry (ETAAS), inductively coupled plasma optical emission spectrometry (ICP-OES), and inductively coupled plasma mass spectrometry (ICP-MS)) used in this field will be documented by works summarized in the following sections. The preparation of MNPs and the modification of MNPs will also be described briefly.

## 3. Preparation of Magnetic Nanoparticles

Magnetic particles with core-shell structures are widely used in MSPE procedures. They contain a magnetic core and a coating. Magnetic composite materials prepared from magnetic particles and other materials (e.g., conventional polymers, molecularly imprinted polymers, and several others) are also described in many MSPE procedures [33].

The selection of suitable magnetic materials plays an important role in achieving good extraction efficiency, enrichment factor, selectivity, and anti-interference ability. Magnetic materials can exhibit different types of magnetism, mainly ferromagnetism, superparamagnetism, diamagnetism, antiferromagnetism, and ferrimagnetism [22]. Magnetic particles composed of Fe, Co, Ni, or their metal oxides/alloys usually possess ferromagnetic or superparamagnetic properties [34]. Upon the application of an external field, magnetic particles tend to act as permanent magnets that form lattices or aggregates depending on the magnetic interaction. Ferromagnetic particles possess permanent magnetism and result in a lattice form when the magnetic field is removed. In contrast, superparamagnetic particles are attracted to magnetic fields; however, magnetic properties do not remain after the elimination of the magnetic field [34]. Among the magnetic particles, iron oxides, such as magnetite (Fe_3_O_4_) and maghemite (*γ*-Fe_2_O_3_), have received considerable attention and become widely used as magnetic cores in magnetic particles.

Another important attribute that affects adsorption efficiency is the size of magnetic particles. In previous literature, descriptions of a wide range of sizes, from nanosized to microsized particles, can be found. Nowadays, magnetic particles with a diameter of 1–100 nm are preferably used. This is because the high surface area of nanoparticles contributes to faster kinetics, thereby improving extraction efficiency.

The methods for the preparation of MNPs include chemical coprecipitation, hydrothermal synthesis, solvothermal synthesis, and sol-gel synthesis [22], as well as the thermal decomposition method, oxidation method, hydrolysis method, microemulsion method, and sonochemical preparation [28].

However, the pure magnetic cores obtained by the methods stated above tend to agglomerate, thus resulting in weakened magnetism when the MNPs are used as adsorbents [22]. To overcome this limitation, suitable surface modification is required.

## 4. Modification of Magnetic Nanoparticles

Due to the instability and vulnerability to oxidations, as well as the propensity to form a cluster, the magnetic core is covered by an external layer. Furthermore, the covering step helps to alter sorbent selectivity [31].

Surface modification methods include physical modification and chemical bonding. The physical modification uses techniques such as surface adsorption, surface deposition, ultraviolet, and plasma radiation. The chemical modification involves a change in the surface state of MNPs through chemical reactions.

The MNP external layer may be designed with inorganic substances (such as silicon dioxide, metallic oxide, and carbon-based materials), organic substances (polymer-based materials and nonpolymer materials), or hybrid materials (such as metal-organic frameworks and MOFs) [28, 31]. MOFs are composed of inorganic subunits (metal ions, clusters, and chains) and organic linkers (nitrogen-containing heterocycle compounds, and carboxylate ligands) [35–37].

Carbonaceous nanomaterials and porous materials are the most popular coating types due to their structure and properties, since they not only improve the stability of MNPs and prevent their oxidation but also offer abundant reaction sites and a large specific surface area [32, 38–43]. Other materials, such as silicon dioxide, metallic oxides, ionic liquids (ILs), chitosan, and surfactants, are also frequently used. The interactions between the target analytes and the abovementioned materials involve electrostatic attraction, hydrogen bonding, *π* − *π* stacking, van der Waals force, hydrophobic force, and metal ionic coordination [22]. However, a complicated matrix may interfere with the adsorbent through nonselective interactions. In such cases, coating MNPs with carefully designed materials, such as molecularly imprinted polymers (MIPs) and aptamers, is a useful method [22]. Alternatively, MNPs can be functionalized with various specific groups, such as carboxyl, hydroxyl, sulfonic acid, amino, mercapto, and chelating functional groups, resulting in favorable selectivity for various target analytes.

## 5. Magnetic Solid Phase Extraction Used for the Separation of Silver Ions and Silver Nanoparticles

**Figure 3:**
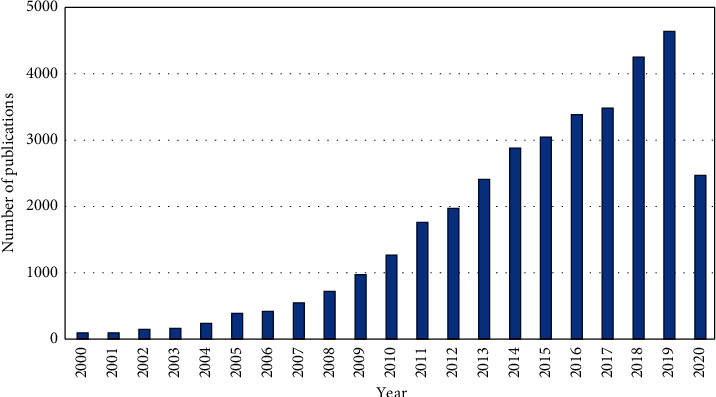
Number of papers published on AgNPs (according to the Web of Science database; between January 2000 and May 2020).

Silver nanoparticles (AgNPs) belong to the group of the most frequently used engineered nanomaterials (Figure 3). This is a result of the well-documented antimicrobial properties of silver and the unique physicochemical properties of AgNPs, including high electrical and thermal conductivity [44]. The widespread range of applications of AgNPs in various consumer products, agriculture, medicine (opto)electronics, and the food industry, as well as many others, increases the chance of their elevated concentrations in the environment [45]. In this sense, AgNPs have become a contaminant of concern for the environment with the possibility of posing risks to human health [44]. Evidence that Ag ions can be easily released from AgNPs [46–50] and the ability of their acute toxic effects on the aquatic environments [34, 51] has led to increased attention on their reliable quantification at ultratrace levels, particularly in aquatic matrices [52]. This challenge is usually achieved by using an efficient separation technique together with a suitable detection method. Among other techniques, MSPE has shown to be a promising alternative for their separation and preconcentration. Examples of various magnetic nanoparticles used in this field can be found in Table 1.

In all the works listed in Table 1, special attention is paid to the synthesis of MNPs used. Preparation procedures described in detail can be found in all of them. In many papers, a thorough characterization of the MNPs is also made. For the characterization, various combinations of the methods, such as IR spectroscopy, scanning electron microscopy (SEM), transmission electron microscopy (TEM), X-ray diffraction (XRD), vibrating sample magnetometry (VSM), elemental analysis, or thermogravimetric analysis, can be found in the published papers. Our interest focuses on the analytical characteristics of the developed procedures.

In the case of procedures for Ag ions, relative standard deviations (RSDs) in the range of 2.7–6.4% were achieved, regardless of the sample analyzed and the detection method used. These RSDs indicate that by using optimized MSPE procedures, highly reproducible results can be obtained.

Most of the published papers describe the use of the procedures for separation/preconcentration of Ag(I) in natural water samples (such as tap, well, river, lake, spring, and seawater samples). In all of them, no Ag(I) was detected and analyses were made after their spiking. The recoveries of the spiked natural water samples, regardless of matrix ion concentrations, were achieved in the range of 89–108%. Wastewater samples, such as electroplating, radiological, and photographic samples, were used for analysis by Jalilian et al. [57]. Very high concentrations of Ag (in the range of 196–266 *μ*g L^−1^) were quantified in these kinds of samples. Even though high concentrations of other components may be present in the analyzed waters, extraction recoveries and RSDs in the range of 99.5–103% and 1.3–1.8% were achieved, respectively. These results demonstrate that efficient procedures were developed, and the methods are suitable for silver analysis, not only in natural water samples but also in wastewater samples.

A further important goal of the proposed MSPE procedures is to improve the limit of detection (LOD). The lowest LOD (2.30 ng L^−1^) was achieved by the combination of MSPE with ETAAS [53]. Despite the LOD achieved, Ag was not detected in the water samples used for analysis.

Food samples were analyzed by Sedghi et al. [55]. They used microwave digestion with concentrated HNO_3_ and total Ag was quantified after its MSPE separation and preconcentration. Among all analyzed samples, Ag was quantified in celery, cabbage, persimmon, and carrot. In these samples, similar concentrations of around 2.0 *μ*g kg^−1^ were found. The recoveries in the spiked samples in the range of 98–101% were achieved.

To assess the applicability of the MSPE method developed by Neyestani et al. [58] to solid samples with high concentrations of many elements, real ore samples were analyzed. The samples were digested using a combination of four acids (HNO_3_, HF, HClO_4_, and HCl) and used for the separation/preconcentration of Ag followed by ICP-OES detection. In this case, the recoveries in the range of 89–92% were achieved.

The last two works mentioned above demonstrate how optimized MSPE procedures using suitable magnetic nanoparticles can serve as an efficient tool to obtain reliable results in the quantification of (ultra)trace Ag, even in highly complicated matrices.

Special attention to this section is dedicated to the applications of magnetic particles to the separation/preconcentration of trace amounts of AgNPs. The use of unmodified and surface-modified magnetic particles in this field has been shown by Mwilu et al. [65]. Citrate-stabilized AgNPs and polyvinylpyrrolidone- (PVP-) stabilized AgNPs were used in their study. In the presence of unmodified magnetic particles (UMPs), selective removal of AgNPs in the presence of Ag ions can be performed. By using glutathione-functionalized magnetic particles (GMPs) and/or dopamine-functionalized magnetic particles (DMPs), Ag ions can be partially coextracted along with AgNPs. Natural water samples (with no detectable Ag) spiked with AgNPs were applied in recovery studies to show the potential of their MNPs for the separation/preconcentration of trace levels of AgNPs in real aqueous matrices.

The use of aged iron oxide magnetic particles (IOMPs) as MSPE adsorbents for speciation analysis of silver sulfide nanoparticles (Ag_2_SNPs) was described by Zhou et al. [64]. They found that IOMPs are excellent adsorbents for selective extraction of silver-containing nanoparticles (AgCNPs), including Ag_2_SNPs, AgNPs, and AgClNPs in the presence of Ag ions. Interestingly enough, it was found that Ag_2_SNPs can be distinguished from the other AgCNPs by sequential elution, thus leading to the validated method for speciation analysis of Ag_2_SNPs in natural water samples.

Tolessa et al. [63] developed magnetic chitosan microspheres (MCMs) as reusable adsorbents for selective separation of AgNPs in the presence of their ionic species. They investigated the effect of size and coating on the extraction efficiencies of AgNPs in detail. AgNPs stabilized with PVP, citrate, and polyvinyl alcohol (PVA) were extracted by the proposed method with similar extraction efficiencies (higher than 91% in all cases). This means that the proposed method has the application potential for separation/preconcentration of AgNPs stabilized with different capping agents with a similar functional group [63].

Zhao et al. [51] prepared poly(1-vinylimidazole) functionalized MNPs (PVIM-MNPs) for the adsorption of AgNPs and Ag ions. With the use of mercaptosuccinic acid as a ligand exchanger, both analytes could be adsorbed on the PVIM-MNPs, and the sequential desorption of Ag ions and AgNPs was achieved by Na_2_S_2_O_3_ and HNO_3_, respectively. On the basis of this, a new approach by the coupling of ligand-assisted MSPE with ETAAS detection was thus proposed for the speciation of AgNPs and Ag ions in environmental water samples.

In each of the published works, natural water samples (with no detectable Ag) spiked with AgNPs were applied in recovery studies to show the potential of the developed MSPE procedures. Regardless of the matrix ion concentrations in the real water samples, high extraction recoveries were achieved in all cases. This fact demonstrates the great application potential of MSPE procedures for AgNPs separation and preconcentration in various environmental water samples.

## 6. Magnetic Solid Phase Extraction Used for the Separation of Gold Ions and Gold Nanoparticles

**Figure 4:**
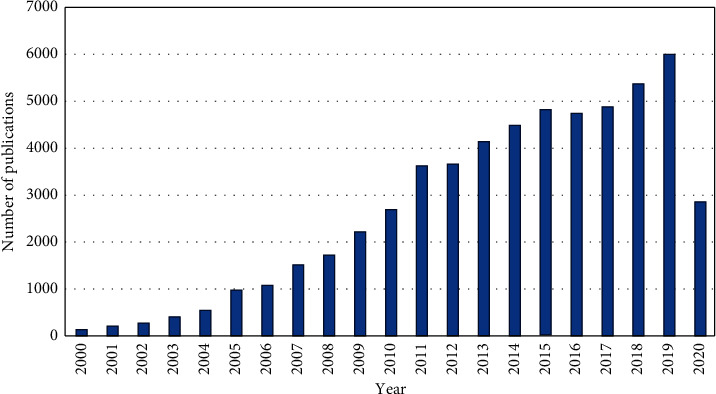
Number of papers published on AuNPs (according to the Web of Science database; between January 2000 and May 2020).

Gold nanoparticles (AuNPs) are extensively used in a wide variety of areas, including electronics and nanotechnology [66]. It stems from their unique optical, physical, and chemical properties [67], as well as controllable synthesis [67, 68]. The ability of AuNPs to be conjugated with many functionalizing agents, such as polymers, surfactants, ligands, dendrimers, drugs, DNA, RNA, proteins, peptides, and oligonucleotides, has led to their extended use in biomedical applications, disease diagnostics, and pharmaceuticals [69–71]. Their extensive use is well documented by many papers published on this topic (Figure 4). This widespread use of AuNPs may result in their increased concentrations in various environmental compartments [72]. However, nanoparticles and their ionic species can coexist and their toxicity may differ; therefore, novel analytical approaches are required to monitor not only the nanoparticles but also their ionic species [73]. Thus, the development of novel methodologies for the separation, preconcentration, and quantification of these emerging pollutants at ultratrace levels is gaining considerable importance [74]. MSPE has shown high potential for the separation/preconcentration of gold ions and AuNPs, which is documented in the works listed in Table 2.

Summarizing the findings from the works listed in Table 2, these general statements can be recorded. Careful verification of the experimental conditions for the synthesis of MNPs was done in all the works. In many of them, a rigorous characterization of the MNPs used is also described. By focusing on analytical characteristics, the following conclusions can be drawn.

In the case of procedures for Au ions, RSDs in the range of 1.1–7.1% were achieved regardless of the sample analyzed and the detection method used. The highest RSDs in the range of 6.1–7.1% were achieved in the case of wastewater samples analysis [57]. Despite that wastewater analyses were performed, no Au was quantified, and MSPE procedures were performed after sample spiking; extraction recoveries in the range of 97–102% were achieved.

Mine samples were analyzed by Ye et al. [76]. They used digestion with aqua regia, and the total content of Au was quantified in the flow arrangement of a microcolumn (packed with crown ether-functionalized magnetic silica nanoparticles) connected with ETAAS. The achieved analytical characteristics, such as high enrichment factor, high sensitivity, and high accuracy, have led to the conclusion that the proposed procedure has great potential for use even for a very complicated sample matrix. Ore samples, as examples of complex matrices, were used by Neyestani et al. [58]. After acid digestion, the samples were utilized for total Au quantification. Excellent LOD of 0.065 *μ*g L^−1^ was achieved in combination with ICP-OES detection. Extraction recoveries for gold ore SRMs were 92 and 102%; real ore samples were spiked and extraction recoveries around 97% were achieved.

In the case of procedures for AuNPs, RSDs around 5% were achieved. These results indicate that reproducible procedures were developed for the studied analyte.

The lowest LODs were obtained by the combination of MSPE with ICP-MS. It was 0.39 ng L^−1^ for Au(III) and 0.31 ng L^−1^ for AuNPs [77]. The authors optimized an MSPE procedure for retaining both analytes using Al^3+^ immobilized Fe_3_O_4_@SiO_2_@iminodiacetic acid as an adsorbent. Their separation was achieved by sequential elution of Au ions and AuNPs with Na_2_S_2_O_3_ and NH_3_.H_2_O, respectively. On this basis, a novel strategy by MSPE with ICP-MS was developed for the speciation of AuNPs and Au ions in environmental water samples.

Slightly higher LODs were achieved by the combination of MSPE with ETAAS. It was 19.7 ng L^−1^ for Au(III) and 19.5 ng L^−1^ for AuNPs [74]. In this case, ascorbic acid enabled the quantitative extraction of both analytes by naked Fe_3_O_4_NPs, whereas a selective extraction of AuNPs was achieved in the presence of sodium thiosulfate. Thus, the authors have published a novel method for AuNPs/total Au speciation based on the combination of MSPE and ETAAS.

With regard to the LODs achieved in the last two works mentioned above, it should also be noted that it is still possible to improve LOD using a higher initial sample volume. If the cost per analysis were a factor in the decision for which detection method to use, ETAAS would still be a cheaper alternative.

## 7. Concluding Remarks

MNPs have been used in various areas due to their unique properties, including a large specific area and simple separation using an external magnetic field. This is well documented by many references in reviews dedicated to the use of MNPs. Here, the great potential of MNPs in MSPE procedures for the separation and preconcentration of AgNPs, AuNPs, and their ionic species is highlighted.

Although the synthesis of MNPs can be laborious and time-consuming, the optimized MSPE procedures can take only a few minutes. In this sense, we are referring to fast extraction procedures. A simple method for sorbent separation with no need for additional procedures, such as centrifugation and/or filtration, is another benefit of MSPE procedures. MNPs can be highly adjustable, and so high selectivity can be achieved even in complex matrices. Another benefit of MSPE, such as fewer interferences (since the majority of sample impurities are diamagnetic), can also be noted. Online coupling of MSPE procedures with detection methods can lead to the automation of the entire process, and the saving of time can be highlighted in such arrangements. Since commonly available spectrometric methods are applied for detection of the analytes, referenced optimized MSPE procedures can be used in any laboratory that has a suitable spectrometric instrumentation. Finally, the possibility of recycling and reusing magnetic adsorbents is also a major benefit.

The extensive use of AgNPs and AuNPs increases the chance of their elevated concentrations in various environmental compartments. In this sense, these nanoparticles have become emerging pollutants. However, the nanoparticles and their ionic species can coexist, and their properties may differ (including toxic effects); therefore, novel analytical approaches are necessary to monitor not only nanoparticles but also their ionic species. The optimized MSPE procedures reviewed in this paper have demonstrated the high potential for the separation and preconcentration of silver ions and gold ions in all types of natural water samples, as well as wastewater samples with high concentrations of coexisting components. Food samples and ore samples were also used for trace analysis of Ag and Au after their separation/preconcentration by the optimized MSPE procedures. High extraction recoveries achieved for these matrices demonstrate that optimized MSPE procedures using suitable MNPs can serve as an efficient tool in obtaining reliable results, even in very complicated matrices.

A key challenge for analytical chemists who work in the field of speciation studies is the reliable quantification of metallic nanoparticles in the presence of their ionic species. The MSPE procedures (in connection with spectrometric methods) reviewed in this paper have shown excellent performance with low LODs, high EFs, high extraction recoveries, and acceptable precision for both AgNPs in the presence of Ag ions and AuNPs in the presence of Au ions.

The reported methods are suitable for fast, cheap, sensitive, and efficient speciation of the analytes in environmental water samples. As a result of the benefits of MSPE procedures, new opportunities for the further development of methodologies based on magnetic separation for selective separation/preconcentration of other metal-containing nanoparticles and their respective ionic species can be expected.

## Figures and Tables

**Table 1:** Magnetic adsorbents used for the separation and preconcentration of Ag ions and AgNPs.

Analyte	Adsorbent	Detection method	*V* (mL)	*m* (mg)	LOD (*μ*g/L)	RSD (%)	Rec. (%)	*Q *_m_ (mg/g)	Ref.
Ag(I)	AC/*γ*-Fe_2_O_3_-BPDM	ETAAS	25	25	2.30^*∗*^	3.5	97–103	32.6	[53]
Ag(I)	TREN-Fe_3_O_4_	ETAAS	100	10	0.80	NR	96–102	97.3	[54]
Ag(I)	Fe_3_O_4_@SiO_2_@SiC_3_H_6_NH_2_	FAAS	150	10	1.10	2.7	98–101	52.3	[55]
Ag(I)	MBT/SDS-ACMNPs	FAAS	10	100	0.56	3.1	96–102	11.6	[56]
Ag(I)	mGO@SiO_2_@PPy-PTh	FAAS	50	19	0.10	2.7	96–103	49.0	[57]
Ag(I)	Mag-GO@MBT/SDS	ICP-OES	50	50	0.045	3.1	89–92	NR	[58]
Ag(I)	AMT-TMSPT-MNPs	ICP-OES	200	50	0.12	5.3	94–103	10.4	[59]
Ag(I)	Fe_3_O_4_@PTh	ICP-OES	100	60	0.20	4.2	89–108	NR	[60]
Ag(I)	DPTH-MNPs	ICP-OES	50	50	0.030	5.6	90–108	9.10	[61]
Ag(I)	DPTH@MGO	ICP-OES	50	50	0.50	3.2	93–106	9.72	[62]
Ag(I)	PVIM-MNPs	ETAAS	50	5	7.50^*∗*^	6.4	80–114	61.4	[51]
AgNPs	PVIM-MNPs	ETAAS	50	5	8.20^*∗*^	7.0	92–122	53.2	[51]
AgNPs^PVP^	MCMs	ICP-MS	20	10	0.023	4.2	85–93	NR	[63]
AgNPs^Citrate^	MCMs	ICP-MS	20	10	0.016	3.4	88–97	NR	[63]
AgNPs^PVA^	MCMs	ICP-MS	20	10	0.021	3.7	88–99	NR	[63]
Ag_2_SNPs	IOMPs	ICP-MS	50	20	0.068	3.0	70–100	NR	[64]

^*∗*^ng/L; *V*: sample volume; *m*: amount of adsorbent; LOD: limit of detection; RSD: relative standard deviation; Rec.: recovery; *Q*_m_: adsorption capacity; Ref.: reference; NR: not reported.

**Table 2:** Magnetic adsorbents used for the separation and preconcentration of Au ions and AuNPs.

Analyte	Adsorbent	Detection method	*V* (mL)	*m* (mg)	LOD (*μ*g/L)	RSD (%)	Rec. (%)	*Q *_m_ (mg/g)	Ref.
Au(III)	mGO@SiO_2_@PPy-PTh	FAAS	50	19	0.10	7.1	97–102	50	[57]
Au(III)	IIP@Fe_3_O_4_	FAAS	20	10	0.43	1.1	95–99	76	[75]
Au(III)	TREN-Fe_3_O_4_	ETAAS	100	10	0.50	NR	91–102	167	[54]
Au(III)	CEMNs	ETAAS	NR	NR	0.16	1.1	95–109	NR	[76]
Au(III)	mag-GO@MBT/SDS	ICP-OES	50	50	0.065	2.5	92–97	NR	[58]
Au(III)	Fe_3_O_4_@PTh	ICP-OES	100	60	2.0	3.3	88–109	NR	[60]
Au(III)	DPTH-MNPs	ICP-OES	50	50	0.62	2.7	92–109	6.20	[61]
Au(III)	DPTH@MGO	ICP-OES	50	50	0.60	2.6	91–104	9.25	[62]
Au(III)	Fe_3_O_4_@SiO_2_@IDA–Al^3+^	ICP-MS	25	20	0.39^*∗*^	3.9	72–99	NR	[77]
AuNPs	Fe_3_O_4_@SiO_2_@IDA–Al^3+^	ICP-MS	25	20	0.31^*∗*^	4.9	73–100	NR	[77]
Au(III)	Fe_3_O_4_NPs	ETAAS	5	0.5	19.7^*∗*^	2.5	53–107	NR	[74]
AuNPs	Fe_3_O_4_NPs	ETAAS	5	0.5	19.5^*∗*^	5.3	85–98	NR	[74]

^*∗*^ng/L; *V*: sample volume; *m*: amount of adsorbent; LOD: limit of detection; RSD: relative standard deviation; Rec.: recovery; *Q*_m_: adsorption capacity; Ref.: reference; NR: not reported.

## Data Availability

No data were used to support this study.

## Abbreviations

AC/**γ**-Fe_2_O_3_-BPDM: Magnetically activated carbon/*γ*-Fe_2_O_3_ modified with 4,4′-bis-(3-phenylthiourea)diphenylmethane
AgClNPs: Silver chloride nanoparticles
AgCNPs: Silver-containing nanoparticles
AgNPs: Silver nanoparticles
Ag_2_SNPs: Silver sulfide nanoparticles
AMT-TMSPT- MNPs: Magnetic nanoparticles coated and modified with 3-(trimethoxysilyl)-1-propanediol and immobilized with 2-amino-5-mercapto-1,3,4-thiadiazole
AuNPs: Gold nanoparticles
CEMNs: Crown ether-coated magnetic nanoparticles
DMPs: Dopamine-functionalized magnetic particles
DPTH@MGO: Magnetic graphene oxide functionalized with 1,5-bis(di-2-pyridil) methylene thiocarbohydrazide
DPTH-MNPs: Magnetic nanoparticles functionalized with 1,5-bis(di-2-pyridil)methylene thiocarbohydrazide
dSPE: Dispersive solid-phase extraction
EF: Enrichment factor
ETAAS: Electrothermal atomic absorption spectrometry
FAAS: Flame atomic absorption spectrometry
Fe_3_O_4_NPs: Naked Fe_3_O_4_ nanoparticles
Fe_3_O_4_@PTh: Polythiophene-coated Fe_3_O_4_
Fe_3_O_4_@SiO_2_@IDA–Al^3+^: Al^3+^-immobilized Fe_3_O_4_@SiO_2_@iminodiacetic acid
Fe_3_O_4_@SiO_2_@SiC_3_H_6_NH_2_: Silica-coated Fe_3_O_4_ modified with poly(2-aminothiophenol)
GMPs: Glutathione-functionalized magnetic particles
ICP-MS: Inductively coupled plasma mass spectrometry
ICP-OES: Inductively coupled plasma optical emission spectrometry
IIP@Fe_3_O_4_: Ion imprinted polymer based on dipyridylamine-coated Fe_3_O_4_ nanoparticles
IOMPs: Iron oxide magnetic particles
IT-SPME: In-tube solid-phase microextraction
LLE: Liquid-liquid extraction
LOD: Limit of detection
mag-GO@MBT/SDS: Magnetized graphene oxide-MnFe_2_O_4_ composite modified with 2-mercaptobenzothiazole-sodium dodecyl sulfate solution
MBT/SDS-ACMNPs: 2-mercaptobenzothiazole/sodium dodecyl sulfate immobilized on alumina-coated magnetite nanoparticles
MCMs: Magnetic chitosan microspheres
mGO@SiO2@PPy-PTh: Magnetic graphene oxide coated with silica and modified with a polypyrrole-polythiophene copolymer
MIPs: Molecularly imprinted polymers
MNPs: Magnetic nanoparticles
MOFs: Metal-organic frameworks
MSPE: Magnetic solid-phase extraction
NR: Not reported
PVA: Polyvinyl alcohol
PVIM-MNPs: Poly(1-vinylimidazole) functionalized magnetic nanoparticles
PVP: Polyvinylpyrrolidone
*Q *_m_: Adsorption capacity
RSD: Relative standard deviation
SBSE: Stir bar sorptive extraction
SEM: Scanning electron microscopy
SPE: Solid-phase extraction
SPME: Solid phase microextraction
TEM: Transmission electron microscopy
TFME: Tin-film microextraction
TREN-Fe_3_O_4_: Tris(2-aminoethyl)amine-functionalized Fe_3_O_4_ nanoparticles
UMPs: Unmodified magnetic particles
VSM: Vibrating sample magnetometry
XRD: X-ray diffraction.
